# Where the Backboned Animals Begin

**Published:** 1883-04

**Authors:** Arabella B. Buckley


Where the Backboned Animals Begin.—There is much uncertainty as to how the backboned or vertebrate animals began; but the best clew we have to the mystery is found in a little, half-transparent creature, about two inches long, which is still to be found living upon the English shores and the Southern Atlantic coast of the United States. This small, insignificant animal is called the "Lancelet," because it is shaped something like the head of a lance; and it is in many ways so imperfect that naturalists believe it to be a degraded form, like the acorn-barnacle—that is to say, that it has probably lost some of the parts which its ancestors once possessed. But, in any case, it is the most simple backboned animal we have, and shows us how the first feeble forms may have lived. Truly, it is only by courtesy that we can call him a backboned animal, for all he has is a cord of gristle, pointed at both ends, which stretches all along the middle of his body above his long, narrow stomach; while above this, again, is another cord containing his nerve-telegraph.

There are large fishes, too, which have this cartilaginous backbone. The young shark has nothing but a rod of gristle or cartilage, and, though he is one of the strongest of sea-animals, *he retains this gristly state of his skeleton throughout his life*; however much he may strengthen it by hard matter, it never becomes true bone.

The first feeble ancestors of the shark and the sturgeon appear at a time when the crustaceans were the most powerful animals in the world, and the huge, lobster-like Pterygotus was the monarch of the seas. The plated-scaled fish which existed at the same time were clumsy creatures, for their skeletons were probably feeble and their armor-like shields were heavy. So, as history went on, they gradually gave way, becoming smaller and rarer, while the more active little shark-like animals gradually grew strong and powerful, and from them are descended the giant sharks of to-day.

The powerful gristly-boned fishes are much excelled in agility by the herring, the salmon, and their other bony companions, which move with much less effort in the water, and so have naturally made their way into all parts of the rivers and seas. But where have they come from? We know very little of their early history, but what little we do know leads us to think that long ago they branched of from the enameled-scaled fish, and struck out a path of their own, to make the most of the watery world.—Arabella B. Buckley, *in Popular Science Monthly for April.*

